# Atomistic Corrective Scheme for Supercell Density Functional Theory Calculations of Charged Defects

**DOI:** 10.1038/s41598-017-02986-5

**Published:** 2017-06-06

**Authors:** Tengfei Cao, Angelo Bongiorno

**Affiliations:** 1 Department of Chemistry, College of Staten Island, Staten Island, NY 10314 USA; 2 Ph.D. Program in Chemistry, The Graduate Center of the City University of New York, New York, NY 10016 USA; 3 Ph.D. Program in Physics, The Graduate Center of the City University of New York, New York, NY 10016 USA

## Abstract

A new method to correct formation energies of charged defects obtained by supercell density-functional calculations is presented and applied to bulk, surface, and low-dimensional systems. The method relies on atomistic models and a polarizable force field to describe a material system and its dielectric properties. The polarizable force field is based on a minimal set of fitting parameters, it accounts for the dielectric screening arising from ions and electrons separately, and it can be easily implemented in any software for atomistic molecular dynamics simulations. This work illustrates both technical aspects and applications of the new corrective scheme. The method is tested on systems in vacuo to validate the energy scheme. It is applied to charged defects in the bulk and at the surface of realistic materials to achieve comparison with published results obtained by using available corrective schemes based on continuum electrostatics treatments. Moreover, to demonstrate its generality, the method is used to correct the formation energy obtained by DFT of a singly negatively charged S vacancy in monolayer, bilayer, trilayer and bulk MoS_2_.

## Introduction

Charged defects pose serious challenges to density functional theory (DFT) calculations relying on the use of supercells^1–3^. The electrostatic energy of a periodic array of charged defects diverges^1, 2^, and to eliminate the singularity, the conventional approach consists in neutralizing the supercell by using a uniform charge background^1, 2^. Periodic DFT calculations rely on this solution to study charged systems and to calculate the formation energy of a charged defect in a dielectric system^3–7^. Unfortunately, the use of supercells and a uniform charge background introduce undesired interactions (between the charged defect and its replicas, and between the charged defect and the uniform background) that are fairly large and turn off slowly with the dimensions of the supercell^1, 2^. To account for these size effects, the formation energy, *E*_*f*_, of a defect carrying a charge *q* is calculated by DFT as follows:

1 $${E}_{f}={E}_{L}^{{\rm{DFT}}}(q)-{E}_{L}^{{\rm{DFT}}}\mathrm{(0)}-{\mu }_{I}-q{\mu }_{e}+{\rm{\Delta }}{E}_{L},$$

where Δ*E*_*L*_ is the corrective energy term canceling the spurious interactions, ${E}_{L}^{{\rm{DFT}}}(q)$ and ${E}_{L}^{{\rm{DFT}}}\mathrm{(0)}$ are the DFT energies of the defected and reference systems, respectively, computed by using supercells of linear dimension *L*, and *μ*_*I*_ and *μ*_*e*_ are the chemical potentials of the ions and electrons forming the defect. In Eq. (1), Δ*E*_*L*_ is equal to *E*_∞_ − *E*_*L*_^1, 3, 6, 8, 9^, that is the difference in electrostatic energy between the isolated defect in the infinite material system, *E*_∞_, and the periodic array of defected supercells of linear dimension *L* compensated by a uniform charge background, *E*_*L*_. Here, we present a new and original method to calculate Δ*E*_*L*_ = *E*_∞_ − *E*_*L*_.

The well-known formula introduced by Makov and Payne is valid and can be used to calculate Δ*E*_*L*_ only in the case of a point-charge defect in an isotropic and homogeneous bulk dielectric material^1^. In recent years, various corrective schemes have been put forward to go beyond the Makov and Payne scheme and calculate Δ*E*_*L*_ in a general situation^7, 8, 10–12^, such as for a charged defect of finite size^3^ in an anisotropic^9^, inhomogeneous^6^, or aperiodic system^6^. All these previous corrective schemes rely on continuum electrostatics, structureless jellium-like models for a defected dielectric system, and either analytical^3^ or numerical^6^ solutions of the Poisson equation. Although all valid, these schemes are limited either in scope to a selected class of defects and systems^1, 3, 9^ or (in our view) by the stringent requirement of using a structureless jellium to represent the defected material system. Here, we present a new, alternative, and general method to correct formation energies of charge defects obtained by DFT. At variance with previous approaches, our method is based on the use of atomistic model structures of a defected material, a simple polarizable force field, and a self-consistent treatment of the dielectric screening arising separately from ions and electrons. It is to be noted that since our method is based on atomistic models and force fields, it can be easily implemented in any software for classical molecular dynamics simulations, and therefore it is readily accessible. In the following, we discuss technical aspects and applications of our method to calculate Δ*E*_*L*_.

## Results and Discussion

### Method description

**Figure 1:**
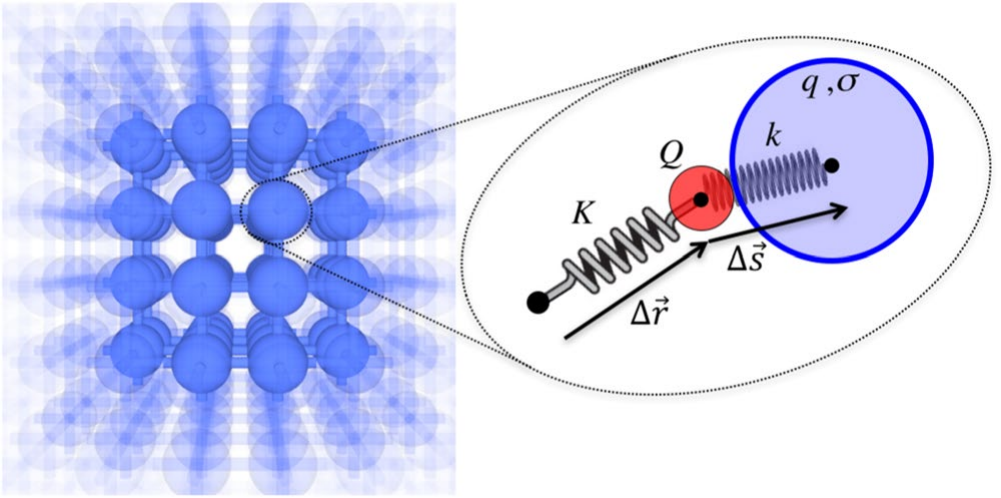
Left panel, ball and stick image of an atomistic model structure. In our scheme, each *atomic* site is described as shown in the 2D schematics enclosed in the dotted ellipse, i.e. an ionic charge, *Q* (red disc), and an electronic charge, *q* (wide blue disc). The ionic charge is connected to an equilibrium position via a harmonic spring *K*, and the electronic charge is connected to the ion via a harmonic spring *k*. The electronic charge can be treated either as a spherical shell or a Gaussian distribution of width *σ*. These parameters are calibrated to reproduce high- and low-frequency dielectric constants of the material system.

Our method relies on the use of periodic atomistic model structures to represent a dielectric material. Each *atomic* site encompasses an ionic charge, *Q*, connected to an equilibrium position by a harmonic spring, *K*, and an electronic charge, *q*, treated either as a spherical shell or a Gaussian charge distribution of width *σ*. Ionic and electronic charges belonging to the same *atomic* site interact only via a harmonic spring, *k*, whereas inter-site electrostatic interactions are calculated by using the Ewald method^13^. In mathematical terms, the potential energy function, *U*, of a periodic supercell encompassing *N* ions and *n* spherical shells takes the following form:

2 $$U={U}_{{\rm{Springs}}}+{U}_{{\rm{Ewald}}},$$

with

3 $${U}_{{\rm{Springs}}}=\frac{1}{2}\sum _{i=1}^{n}{k}_{i}{\rm{\Delta }}{s}_{i}^{2}+\frac{1}{2}\sum _{j=1}^{N}{K}_{j}{\rm{\Delta }}{r}_{j}^{2},$$

and

4 $$\begin{array}{rcl}{U}_{{\rm{Ewald}}} & = & \frac{1}{2}\sum _{{\bf{n}}}\sum _{i}^{N+n}\sum _{j\ne i}^{N+n}\frac{{\tilde{q}}_{i}{\tilde{q}}_{j}}{|{\tilde{{\bf{r}}}}_{i}-{\tilde{{\bf{r}}}}_{j}-{{\bf{R}}}_{{\bf{n}}}|}{\rm{erfc}}(\frac{{\tilde{{\bf{r}}}}_{i}-{\tilde{{\bf{r}}}}_{j}-{{\bf{R}}}_{{\bf{n}}}}{\sqrt{2}{\sigma }_{ew}})\\  &  & ++\frac{2\pi }{V}\sum _{{\bf{G}}\ne 0}\frac{\exp (-\frac{{\sigma }_{{\rm{ew}}}^{2}{{\rm{G}}}^{2}}{2})}{{G}^{2}}{|S({\bf{G}})|}^{2}-\frac{1}{\sqrt{2\pi }{\sigma }_{ew}}\sum _{i}^{N+n}{\tilde{q}}_{i}^{2}\\  &  & +-\sum _{i}^{n}\frac{{q}_{i}{Q}_{i}}{{\rm{\Delta }}{s}_{i}}{\rm{erf}}(\frac{{\rm{\Delta }}{s}_{i}}{\sqrt{2}{\sigma }_{ew}}),\end{array}$$

where, in the equations above, *V* is the volume of the supercell, **n** and **R**_**n**_ are indexes and corresponding lattice vector of the direct space of supercells, **G** is a vector of the reciprocal space, $\tilde{q}$ indicates either ionic (*Q*) or electronic (*q*) charges, $\tilde{{\bf{r}}}$ refers to the vector position of either an ion (**r**) or an electronic charge (*s*), *σ*_*ew*_ is the parameter controlling the convergence of the Ewald sums, Δ*r* refers to the displacement of an ion from its equilibrium position, and Δ*s* indicates the distance between ion and the respective electronic charge (see Fig. 1). In Eq. (4), *S*(**G**) is the structure factor of the ionic and electronic charges, the first sum excludes the interactions between ions and electronic charges belonging to same sites, and the last sum is used to eliminate these same interactions from the sum in the reciprocal space. In case of electronic charges treated as Gaussian charge distributions, Eq. (2) includes the following additional term:

5 $$\begin{array}{rcl}{U}_{{\rm{Gaussians}}} & = & -\sum _{{\bf{n}}}\sum _{i}^{n}\sum _{j}^{N}\frac{{q}_{i}{Q}_{j}}{|{{\bf{s}}}_{i}-{{\bf{r}}}_{j}-{{\bf{R}}}_{{\bf{n}}}|}{\rm{erfc}}(\frac{{{\bf{S}}}_{i}-{{\bf{r}}}_{j}-{{\bf{R}}}_{{\bf{n}}}}{\sqrt{2}{\sigma }_{i}})\\  &  & +-\sum _{{\bf{n}}}\sum _{i}^{n}\sum _{j\ne i}^{n}\frac{{q}_{i}{q}_{j}}{|{{\bf{s}}}_{i}-{{\bf{s}}}_{j}-{{\bf{R}}}_{{\bf{n}}}|}{\rm{erfc}}(\frac{{{\bf{s}}}_{i}-{{\bf{s}}}_{j}-{{\bf{R}}}_{{\bf{n}}}}{\sqrt{\mathrm{2(}{\sigma }_{i}^{2}+{\sigma }_{j}^{2})}}),\end{array}$$

where the *σ*_*i*_’s correspond to the widths of the Gaussian distributions assigned to the electronic charge.

To determine equilibrium displacements of both ions and electronic charges (due to the presence of either a static electric field or a charge defect), we assign fictitious masses to both ionic and electronic charges and we use conventional damped molecular dynamics techniques^13^ to determine the ground state energy and dielectric displacements of the supercell. Periodic model structures incorporating a charged defect are neutralized by using a uniform charge background, and to estimate the correction energy Δ*E*_*L*_ = *E*_∞_ − *E*_*L*_ to be used in Eq. (1), the polarizable energy scheme in Eqs (2) and (5) is used to calculate *E*_*L*_ and, by extrapolation, *E*_∞_. Unless otherwise specified, $L=\sqrt[3]{V}$.

The parameters {*K*, *Q*} and {*k*, *q*, *σ*} for each *atomic* site require to be calibrated to reproduce the high- and low-frequency dielectric constants of the material system. In case of homogeneous (periodic and simple aperiodic) systems, all cationic/anionic sites are equivalent, and thus our scheme requires to determine the values of only a few parameters. In this case, the fitting procedure involves three simple steps. First, selecting the value for the ionic and electronic charges, and optionally of the width of the Gaussian distributions; this task is easily accomplished by relying on chemical intuition and formal oxidation numbers. Second, using the Clausius-Mossotti relationships to obtain tentative values for the harmonic spring constants *k* and *K*. Third, carrying out a few calculations with the polarizable energy scheme to refine these tentative values and obtain the optimal spring constants yielding the desired values for both the high- and low-frequency dielectric constants of the material system of interest. In the general case of non-equivalent sites, such as for complex surfaces or interfaces, or multicomponent systems, the fitting procedure may, in principle, become involved. In this case, however, DFT calculations of dielectric-constant spatial profiles may facilitate the task, as it has been recently shown for a corrective scheme based on solving the Poisson equation for continuum models of inhomogeneous materials^6^.

### Applications to model systems

We first apply our method to the following charge distributions in cubic supercells of vacuum: (i) a unit point charge, (ii) a unit charge distributed according to a Gaussian distribution of width 4 *a*_0_, and (iii) two Gaussian charge distributions of width 1 *a*_0_ placed on the diagonal of the supercell at a distance of $10\sqrt{3}{a}_{0}$. In all cases, we use our scheme to calculate the energy of the array of charges interacting with a compensating uniform charge background.

**Figure 2:**
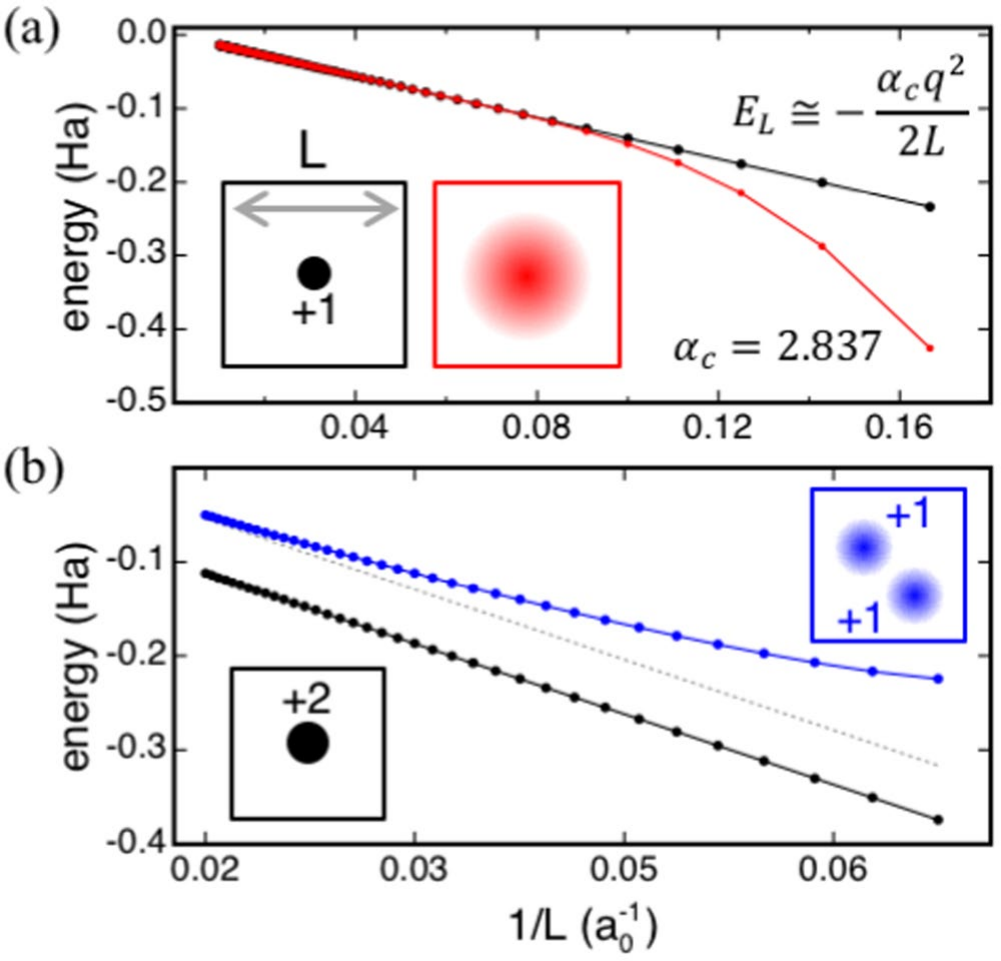
(**a**) Energy vs. *L* ^−1^ of (black discs) a cubic array of unit point charges and (red discs) unit Gaussian charges of width 4 *a*_0_. (**b**) Energy vs. *L* ^−1^ of (black discs) a point charge with *q* = +2 and (blue discs) two unit Gaussian charges of width 1 *a*_0_ separated by a distance of $10\sqrt{3}{a}_{0}$. For clarity, schematics of the charged periodic systems, as well as the Makov-Payne formula and the Madelung constant obtained by fitting the energy values at large *L* are shown as insets.

The energy of a cubic array of point charges scales linearly with *L* ^−1^, according to the scaling function shown as inset in Fig. 2, with a Madelung constant derived by fitting our data in excellent agreement with published results^10^. Our scheme yields also the correct behavior for the energy of an array of Gaussian charges (Fig. 2). In this case, in addition to terms scaling as *L* ^−1^, the energy depends also on terms scaling as *L* ^−3^, accounting for both the size and quadrupole moment of the charge distribution in a supercell (Fig. 2)^1, 10^. These results demonstrate that our method encompasses correction schemes relying on analytical formulas, applicable to charged defects of finite size in homogeneous bulk systems^1, 3^.

To illustrate key technical aspects of our method, we consider a charged defect in a fictitious material with a static dielectric permittivity of 4. The material is described by using cubic lattices with a spacing of 2 Å. Each site of the lattice encompasses an immobile ion carrying a positive unit charge, and an electronic spherical shell carrying a negative unit charge, connected to the ion via a harmonic spring *k*. To determine the value of *k* yielding the desired permittivity, we use the Clausius-Mossotti relation to estimate a tentative value (in this case *k* = 15.1 eV/Å^2^), and then we perform a few trial-and-error calculations to determine the optimal value *k* = 11.0 eV/Å^2^. We use this model dielectric material to calculate values of *E*_∞_ − *E*_*L*_ with supercells hosting a unit charge defect in the center of a cubic interstice of the lattice.

**Figure 3:**
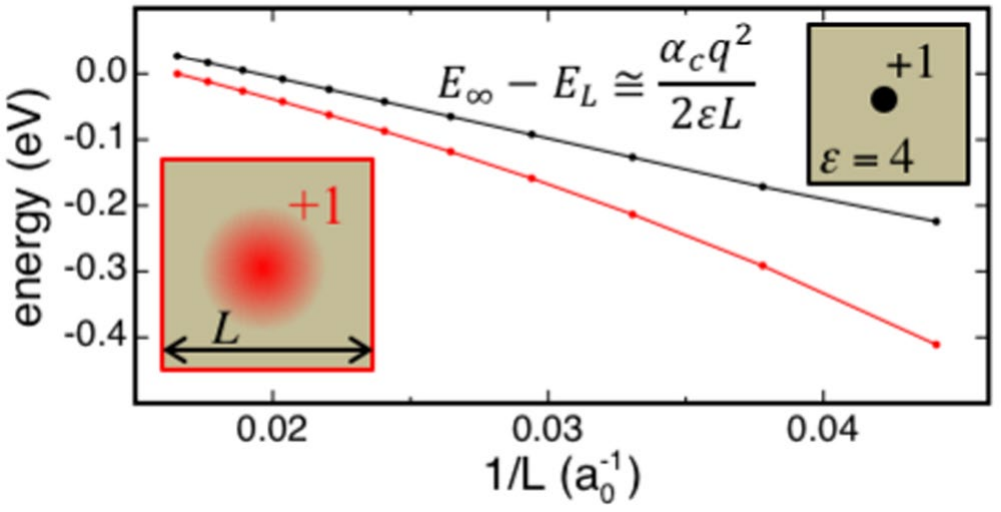
Energy vs. *L* ^−1^ of a cubic array of (black discs) unit point charges and (red discs) a unit Gaussian charge of width 2 Å in a model dielectric material with a permittivity of 4. For clarity, schematics of the defected supercells and energy scaling function are shown as insets.

Figure 3 shows that, in the case of a point charge defect, the energy scales linearly with *L* ^−1^, with a prefactor proportional to the Madelung constant of a cubic lattice and inversely proportional to the permittivity of the material. In the case of a defect charge distributed according to a Gaussian distribution of width 2 Å, our calculations show that in supercells of small to moderate size, the charge is only partially screened by the dielectric material, and thus that the linear scaling, or else the continuum limit, is approached only at large *L* (Fig. 3), when dielectric displacements are not constrained by periodic boundary conditions. These results demonstrate that, thanks to the atomistic representation and the self-consistent treatment of dielectric screening, our method accounts, at variance with previous schemes, for size effects associated with the screening of charged defects of finite size.

### Applications to realistic systems

To show applications to realistic materials, we consider a Cl^−^ vacancy defect in the bulk and at the (100) surface of NaCl^6, 14^, and a doubly negatively charged B_*N*_ antisite defect in the bulk of the hexagonal form of BN^9^. Here we use our method to calculate the energy correction, *E*_∞_ − *E*_*L*_, and achieve comparison with recent computational studies of these defects^6, 9, 14^. To represent NaCl, we use rock-salt-type lattices, whereas for h-BN, we use layered atomistic structures with the AA′ stacking pattern. In both cases, we use experimental lattice constants. With the parameters reported in Table 1, our energy scheme yields, in agreement with experimental and DFT results, a dielectric constant of 5.9 (${\epsilon }_{\infty }=2.4$) for NaCl, and of 6.6 and 3.5 for the components of the dielectric tensor perpendicular and parallel to the *c* axis of h-BN, respectively (${\epsilon }_{\infty }^{\perp }=4.3$ and ${\epsilon }_{\infty }^{\parallel }=2.6$)^9, 14^. To describe the charged defects, we remove a Cl^−^ site from the lattice of bulk models, and at the terminal layer of surface models of NaCl. In the case of the ${{\rm{B}}}_{N}^{2-}$ antisite defect in h-BN, we replace a N site with an *atomic* site carrying an ionic charge of 0.25*e* and an electronic charge of −3.25*e*, distributed according to a Gaussian distribution of width 1.5 Å; both charges are fixed at the equilibrium position.

**Table 1:** Anionic and cationic parameters used in our atomistic polarizable energy scheme to reproduce high- and low-frequency dielectric constants of NaCl, h-BN, and monolayer MoS_2_.

Material	Cation, (*Q*, *K*); (*q*, *k*, *σ*)	Anion, (*Q*, *K*); (*q*, *k*, *σ*)
NaCl	Na, (1.0, ∞); (0, −, −)	Cl, (0.0, 2.95); (−1.0, 4.00, −)
h-BN	B, (1.0, 5.50); (0, −, −)	N, (0.25, 6.0); (−1.25, 6.0, 1.0)
1L-MoS_2_	Mo, (0.0, ∞); (0, −, −)	S, (2.0, ∞); (−2.0, 12.4, 0.5)

Harmonic spring constants, *K* and *k*, are expressed in eV/Å^2^, the Gaussian width, *σ*, in Å, and atomic and electronic charges, *Q* and *q*, in electron charge unit.

**Figure 4:**
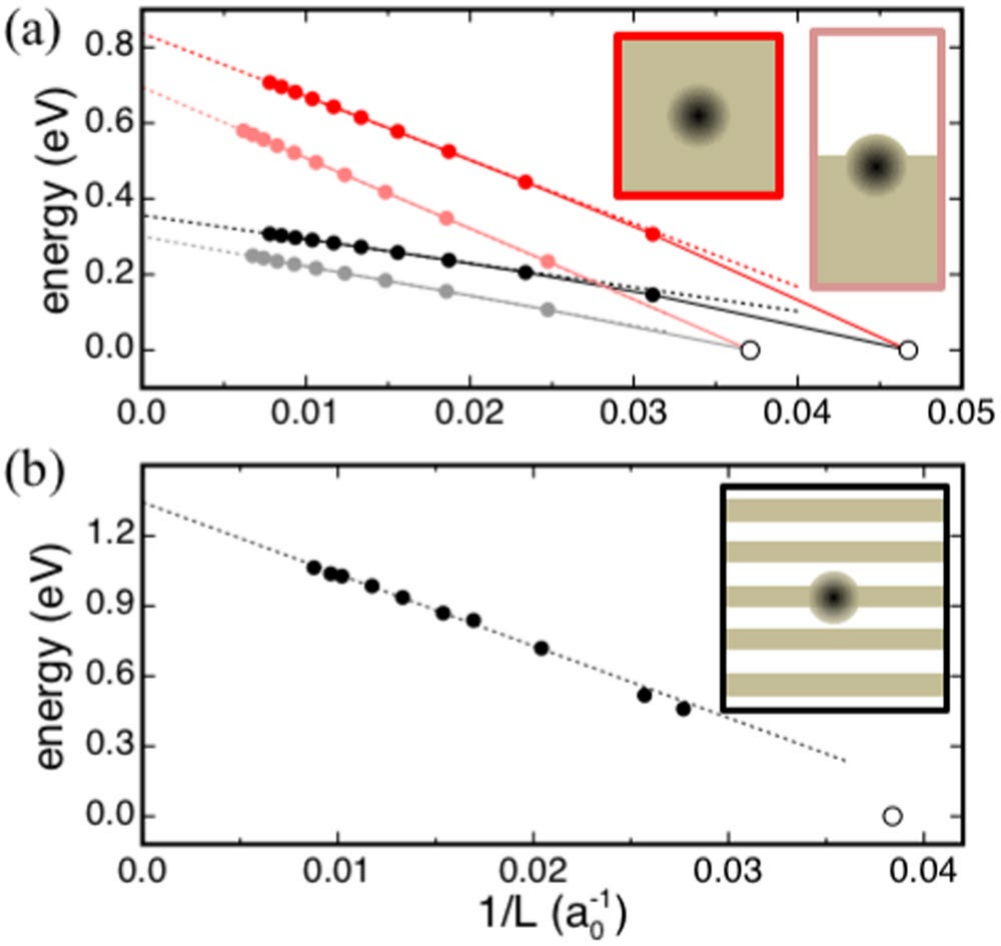
(**a**) Energy vs. *L* ^−1^ for a Cl^−^ vacancy defect in (red) rigid and (black) fully polarizable lattice models of (vivid colors) bulk NaCl and (pale colors) the NaCl(001) surface. Supercells of bulk NaCl are cubic, whereas surface models consist of tetragonal supercells with *c* = 2*a* and equal parts of material and vacuum along the *c* axis. Energy values are refereed to that of a defected supercell with a dimension along the *a* axis equal to 2*l*_0_, where *l*_0_ is the experimental lattice constant. (**b**) Energy vs. *L* ^−1^ for a ${{\rm{B}}}_{N}^{-2}$ antisite defect in orthorhombic model structures of h-BN. Discs show energy values calculated by using our polarizable energy scheme, and dotted lines show linear interpolations. Insets show schematics of the defected materials systems.

Recently, Chen *et al*. used a periodic DFT scheme to calculate the formation energy of a Cl^−^ vacancy defect in rigid supercells of NaCl, i.e. consisting of immobile ions^14^. Based entirely on DFT, this study obtained a value for *E*_∞_ − *E*_*L*_ of about 0.7 eV, with *L* equal to twice the lattice constant, *l*_0_, of NaCl^14^. For the same value of *L*, our method yields an energy correction of about 0.8 eV (Fig. 4), in agreement with the result obtained by extrapolating a few energy values calculated by DFT using supercells with *L* up to a value of 5*l*_0_^14^. Also, our method yields an energy correction of 0.7 eV for a Cl^−^ vacancy defect at the (001) surface of rigid NaCl crystals (with $L=\sqrt[3]{2\times 2\times 4}\,{l}_{0}$). This result is in agreement with the energy correction of about 0.6 eV obtained by solving the Poisson equation for continuum models of the defected surface^6^.

Figure 4 shows the energy values obtained for a Cl^−^ vacancy defect in both rigid and fully polarizable crystals, i.e. in which both ions and electronic shells participate in screening the charged defect. In both cases, and for both bulk and surface, at large *L* the energy scales linearly with *L* ^−1^, with a scaling function proportional to the Madelung constant of the lattice of supercells and inversely proportional to the static permittivity of the periodic system (Fig. 4). By fitting the energy values of the defected surface, we find, as expected, a Madelung constant equal to 2.275, and a dielectric constant equal to $({\epsilon }_{0}+\mathrm{1)/2}$, corresponding to the Madelung constant and permittivity of an array of tetragonal supercells, with *c* = 2*a* and encompassing equal parts of dielectric (${\epsilon }_{0}=5.9$) and vacuum (Fig. 4).

A good agreement with a recent DFT study^9^ is also obtained in the case of the ${{\rm{B}}}_{N}^{2-}$ defect in h-BN. In fact, our method gives *E*_∞_ − *E*_*L*_ = 0.85 eV to correct a formation energy obtained by DFT using a supercell of 8 × 8 × 3 unit cells, in close agreement with the energy value of about 0.75 eV obtained by Kumagai and Oba^9^. Also, we remark that, at variance with previous cases, the linear scaling function interpolating the energy values at large *L* (Fig. 4) is not related trivially to the symmetry and dielectric constant of the material. In case of anisotropic materials such as h-BN, the numerical approach is the only viable strategy to estimate *E*_∞_ − *E*_*L*_^9^. Overall, these results demonstrate that our method is sound and applicable to a variety of defected systems, including aperiodic and anisotropic materials.

### Application to a charged defect in a low-dimensional material

To demonstrate the generality of our method, we consider also the case of a charged defect in low-dimensional materials. In particular, we calculate the energy correction resulting from periodic DFT calculations of a singly negatively charged S vacancy in monolayer, bilayer, and tri-layer (and for completeness also in bulk) MoS_2_^15, 16^.

Our periodic DFT calculations^17^ are carried out using normconserving pseudopotentials^18^ for both Mo and S, a generalized gradient approximation for the exchange and correlation energy^19^, a plane-wave energy-cutoff of 80 Ry, and a semi-empirical corrective scheme to account for London dispersion interactions^20^. To describe the MoS_2_ systems, we use orthorhombic supercells and atomic positions in accord to the 2H crystalline phase of MoS_2_ with a AB layer stacking pattern. Single layers include 30 MoS_2_ formula units, and 2D films are separated by vacuum regions of about 12 Å. We use DFT to calculate structural, dielectric, and electronic properties, as well as the formation energy of the neutral and singly negatively charged S vacancy defect. Lattice parameters of monolayer and bulk MoS_2_ are found ~3% larger than experimental values, and in the case of bilayer and trilayer MoS_2_, the intra-layer spacing between S planes is found to be 3.17 Å, and the inter-layer separation between Mo planes is found to be 6.21 Å. Overall, structural, dielectric, and electronic properties computed in this study are in agreement with recent DFT study of the MoS_2_ systems^16^. To calculate formation energies, we use half the energy of molecular S_2_ and a Fermi level equal to the valence-band edge as chemical potentials for S and electrons, respectively. Relevant DFT results are reported in Table 2.

**Table 2:** Results obtained from DFT calculations for the dielectric constants (perpendicular, ${\epsilon }_{\perp }$, and parallel, ${\epsilon }_{\parallel }$, to the layers), work function (Φ), and formation energies of the neutral and singly negatively S vacancy in monolayer (1L), bilayer (2L), tri-layer (3L), and bulk MoS_2_.

MoS_2_	*ε*_⊥_	${{\boldsymbol{\epsilon }}}_{{\boldsymbol{\parallel }}}$	Φ	E_*f*_ (${{\bf{V}}}_{{\boldsymbol{S}}}^{{\bf{0}}}$)	E_*f*_ (${{\bf{V}}}_{{\boldsymbol{S}}}^{-{\bf{1}}}$)	E_∞_-E_*L*_
1L	1.9	14.1	6.1	1.65	3.29	0.30
2L	2.6	14.8	5.5	1.87	2.67	0.17
3L	2.9	14.8	5.3	1.88	2.61	0.08
bulk	6.0	14.7	—	1.92	2.47	0.10

The last column reports the energy corrections for the formation energies of the charged defects obtained by using the method presented in this work. Energy values are in eV.

**Figure 5:**
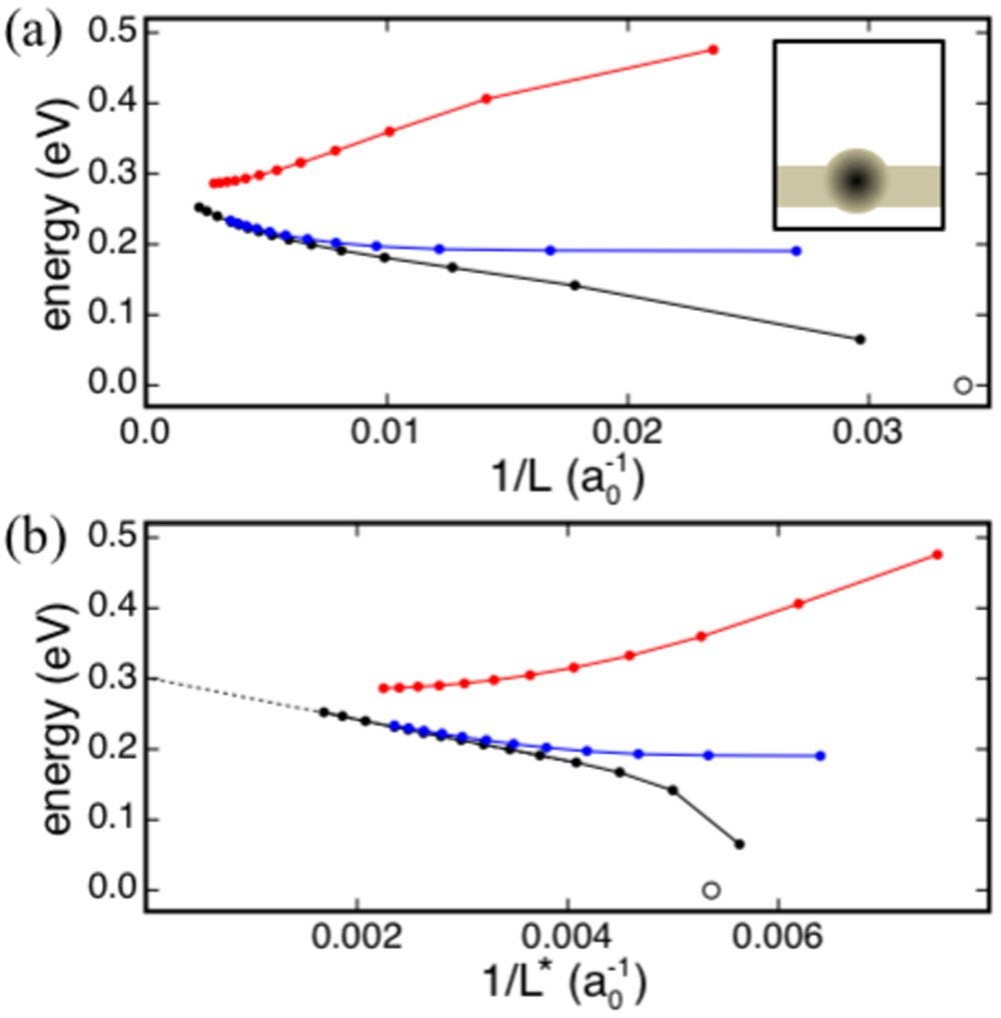
(**a**) Energy vs. *L* ^−1^ for a singly negatively charged S vacancy in monolayer MoS_2_ calculated by using orthorhombic supercells with edges parallel to the monolayer equal to *l*_*x*_ and ${l}_{y}=\sqrt{3}\mathrm{/2}{l}_{x}$, and with a perpendicular edge equal to (red) *l*_*z*_ = 2*l*_*x*_, (black) *l*_*z*_ = *l*_*x*_, or (blue) *l*_*z*_ = *l*_*x*_ + *d* (*d* = 6.1 Å is an approximate value for the monolayer thickness). Energy values are obtained with supercells of increasing *l*_*x*_ and plotted against $L=\sqrt{{l}_{x}{l}_{y}{l}_{z}}$. (**b**) Same as (**a**), with energy values plotted versus the rescaled linear dimension ${L}^{\ast }={\epsilon }_{SC}L$, with ${\epsilon }_{SC}=({\epsilon }_{0}^{\parallel }-1)d/{l}_{z}+1$, where ${\epsilon }_{0}^{\parallel }$ is the component of the dielectric tensor parallel to the monolayer. Discs show energy values obtained by using our energy scheme, and the dotted line shows the linear interpolation of energy values at large *L* obtained by using supercells with *l*_*z*_ = *l*_*x*_. Energy values are referred to that one obtained with the same supercell used to calculate the defect formation energy by DFT. The inset shows a schematics of the defected 2D material.

With the parameters in Table 1, our polarizable energy scheme gives, in agreement with our DFT calculations (Table 2), an in-plane and out-of-plane dielectric constant of 14.1 and 2.0, respectively, for monolayer MoS_2_, and values of 14.5 and 2.2 for bilayer MoS_2_, 14.7 and 2.5 for the tri-layer film, and 15.0 and 6.6 for the bulk phase. This polarizable energy scheme and atomistic model structures of the MoS_2_ systems are thus used to estimate the energy corrections for the formation energies of the charged S vacancy defect obtained by DFT (Table 2). In these calculations, the defect is described by using a negative unit charge in the center of a triangular interstice formed by nearest neighboring Mo ions. Point- or Gaussian-like (with a width of 1 Å) distributions for the unit charge yield results differing by only 0.02 eV. Figure 5 shows the results obtained in the case of monolayer MoS_2_; similar energy vs. 1/*L* curves are obtained also in the case of bilayer and trilayer MoS_2_. The correction energies obtained by extrapolation of the energy values to infinite volumes are shown in Table 2.

It has to be noted that due to the low dimensionality of the material, the defect energy in Fig. 5 scales non-trivially with *L* ^−1^. In the present case, we find that extrapolation of the energy values to large volumes is aided by introducing a rescaled linear dimension *L* ^*^, related to *L*, thickness and in-plane dielectric constant of the 2D MoS_2_ systems as described in the caption of Fig. 5. In agreement with recent DFT studies^8, 11, 12, 16^, these results demonstrate that the task of calculating the formation energy of a charged defect in a low-dimensional material cannot be accomplished by using only DFT. A numerical scheme needs to be used to estimate Δ*E*_*L*_ = *E*_∞_ − *E*_*L*_, which in the case of a singly negatively charged S vacancy in monolayer MoS_2_ amounts to about 10% of the energy value obtained by DFT (Table 2).

## Conclusion

We have introduced and applied a novel general method to correct the energy of charged defects obtained by DFT. At variance with previous methods, our approach is based on a polarizable energy scheme, atomistic representations, and a self-consistent treatment of the dielectric screening. The force field is based on a minimal set of fitting parameters, which can be easily calibrated by relying on either experimental data or, in case of more complex cases such as aperiodic or multicomponent systems, polarizability profiles derived from DFT calculations^6^. Being based on atomistic models, force fields, and Ewald sums, our method can be easily implemented in any available software for molecular dynamics simulations of materials or biological systems. Therefore, besides being new and general, our method is readily accessible to the community interested in DFT calculations and charged defects.

## References

[1] Makov G, Payne MC (1995). Periodic boundary conditions in *ab initio* calculations. Phys. Rev. B.

[2] Leslie M, Gillan NJ (1985). The energy and elastic dipole tensor of defects in ionic crystals calculated by the supercell method. Journal of Physics C: Solid State Physics.

[3] Freysoldt C, Neugebauer J, Van de Walle CG (2009). Fully *Ab Initio* finite-size corrections for charged-defect supercell calculations. Phys. Rev. Lett..

[4] Castleton CWM, Höglund A, Mirbt S (2006). Managing the supercell approximation for charged defects in semiconductors: Finite-size scaling, charge correction factors, the band-gap problem, and the *ab initio* dielectric constant. Phys. Rev. B.

[5] Lany S, Zunger A (2008). Assessment of correction methods for the band-gap problem and for finite-size effects in supercell defect calculations: Case studies for zno and gaas. Phys. Rev. B.

[6] Komsa H-P, Pasquarello A (2013). Finite-size supercell correction for charged defects at surfaces and interfaces. Phys. Rev. Lett..

[7] Komsa H-P, Rantala TT, Pasquarello A (2012). Finite-size supercell correction schemes for charged defect calculations. Phys. Rev. B.

[8] Wang D (2015). Determination of formation and ionization energies of charged defects in two-dimensional materials. Phys. Rev. Lett..

[9] Kumagai Y, Oba F (2014). Electrostatics-based finite-size corrections for first-principles point defect calculations. Phys. Rev. B.

[10] Dabo I, Kozinsky B, Singh-Miller NE, Marzari N (2008). Electrostatics in periodic boundary conditions and real-space corrections. Phys. Rev. B.

[11] Chan T-L, Zhang SB, Chelikowsky JR (2011). Charged dopants in semiconductor nanowires under partially periodic boundary conditions. Phys. Rev. B.

[12] Komsa H-P, Berseneva N, Krasheninnikov AV, Nieminen RM (2014). Charged point defects in the flatland: Accurate formation energy calculations in two-dimensional materials. Phys. Rev. X.

[13] Allen, M. & Tildesley, D. *Computer Simulation of Liquids* (Oxford: Clarendon Pr, 1987).

[14] Chen W, Tegenkamp C, Pfnür H, Bredow T (2010). Color centers in nacl by hybrid functionals. Phys. Rev. B.

[15] Komsa H-P, Krasheninnikov AV (2015). Native defects in bulk and monolayer mos_2_ from first principles. Phys. Rev. B.

[16] Noh J-Y, Kim H, Kim Y-S (2014). Stability and electronic structures of native defects in single-layer mos_2_. Phys. Rev. B.

[17] Giannozzi, P. *et al*. Quantum espresso: a modular and open-source software project for quantum simulations of materials. *Journal of Physics: Condensed Matter* **21**, 395502 (19pp) (2009). 10.1088/0953-8984/21/39/395502 21832390

[18] Troullier N, Martins JL (1991). Efficient pseudopotentials for plane-wave calculations. Phys. Rev. B.

[19] Perdew JP, Burke K, Ernzerhof M (1996). Generalized gradient approximation made simple. Phys. Rev. Lett..

[20] Grimme S (2006). Semiempirical gga-type density functional constructed with a long-range dispersion correction. Journal of Computational Chemistry.

